# Topologies on Superspaces of TVS-Cone Metric Spaces

**DOI:** 10.1155/2014/640323

**Published:** 2014-01-22

**Authors:** Xun Ge, Shou Lin

**Affiliations:** ^1^School of Mathematical Sciences, Soochow University, Suzhou 215006, China; ^2^Department of Mathematics, Ningde Normal University, Fujian 352100, China; ^3^Department of Mathematics, Zhangzhou Normal University, Zhangzhou 363000, China

## Abstract

This paper investigates superspaces *𝒫*_0_(*X*) and *𝒦*_0_(*X*) of a tvs-cone metric space (*X*, *d*), where *𝒫*_0_(*X*) and *𝒦*_0_(*X*) are the space consisting of nonempty subsets of *X* and the space consisting of nonempty compact subsets of *X*, respectively. The purpose of this paper is to establish some relationships between the lower topology and the lower tvs-cone hemimetric topology (resp., the upper topology and the upper tvs-cone hemimetric topology to the Vietoris topology and the Hausdorff tvs-cone hemimetric topology) on *𝒫*_0_(*X*) and *𝒦*_0_(*X*), which makes it possible to generalize some results of superspaces from metric spaces to tvs-cone metric spaces.

## 1. Introduction

Ordered normed spaces and cones have many applications in applied mathematics, for instance, in using Newton's approximation method [1–4] and in optimization theory [5]. By using an ordered Banach space instead of the set of real numbers as the codomain for a metric, *K*-metric and *K*-normed spaces were introduced in the mid-20th century ([2], see also [3, 4, 6]). Huang and Zhang [7] reintroduced such spaces under the name of cone metric spaces. In some results about metric spaces, can metric spaces be relaxed to cone metric spaces? This is an interesting question and many relevant results have been obtained (see [7–11], e.g.). Recently, Khani and Pourmahdian [9] proved that each cone metric space is metrizable, which shows that some improvements by relaxing metric spaces to cone metric spaces are trivial. This leads us to discuss more general cone metric spaces, which further addresses the relationship between metric topology and geometry. In our discussion, it is interesting to consider certain topological groups in place of Banach spaces in the definition of cone metric spaces, which can serve as a topic for further studies [9]. In fact, Du [12] introduced and investigated tvs-cone metric spaces by replacing Banach spaces with topological vector spaces in the definition of cone metric spaces. In the past years, tvs-cone metric spaces have aroused many mathematical scholars' interests and some interesting results have been obtained (see [12–15], e.g.). As an important result for tvs-cone metric spaces, it is proved that each tvs-cone metric space is metrizable ([13, 14], e.g.), which makes it meaningless to research topological properties of tvs-cone metric spaces. However, we notice that some results related to nontopological properties, for example, metric properties (including hemimetric properties), are not direct consequences of known theorems. In particular, we are interested in tvs-cone hemimetric properties on superspaces of tvs-cone metric spaces.

In fact, superspace is an important concept in topological spaces theory. For superspaces of metric spaces, we often deal with six topologies: the lower topology, the lower hemimetric topology, the upper topology, the upper hemimetric topology, the Vietoris topology, and the Hausdorff hemimetric topology. What relationships are there among these topologies? It is an interesting question. Let *𝒫*_0_(*X*) and *𝒦*_0_(*X*) be the space consisting of nonempty subsets of *X* and the space consisting of nonempty compact subsets of *X*, respectively. And then the following two theorems are well known (see [16], e.g.).

**Theorem 1**

Let (*X*, *d*) be a metric space and let *𝒞* be a subset of *𝒫*_0_(*X*). Then the following hold.

1. If *𝒞* is open in the lower topology on *𝒫*_0_(*X*), then *𝒞* is open in the lower hemimetric topology on *𝒫*_0_(*X*).
2. If *𝒞* is open in the upper hemimetric topology on *𝒫*_0_(*X*), then *𝒞* is open in the upper topology on *𝒫*_0_(*X*).

**Theorem 2**

Let (*X*, *d*) be a metric space. Then the following hold.

1. The lower topology and the lower hemimetric topology coincide on *𝒦*_0_(*X*).
2. The upper topology and the upper hemimetric topology coincide on *𝒦*_0_(*X*).
3. The Vietoris topology and the Hausdorff hemimetric topology coincide on *𝒦*_0_(*X*).

As a concrete exploration for tvs-cone metric properties, the following question arises from Theorems 1 and 2 naturally.

**Question 1**

Can Theorems 1 and 2 be generalized from metric space to tvs-cone metric space?

This paper investigates superspaces *𝒫*_0_(*X*) and *𝒦*_0_(*X*) of a tvs-cone metric space (*X*, *d*). The purpose of this paper is to establish some relationships between the lower topology and the lower tvs-cone hemimetric topology (resp., the upper topology and the upper tvs-cone hemimetric topology and the Vietoris topology and the Hausdorff tvs-cone hemimetric topology) on *𝒫*_0_(*X*) and *𝒦*_0_(*X*), respectively. These results answer Question 1 affirmatively and make it possible to generalize the discussions for superspaces from metric spaces to tvs-cone metric spaces.

Throughout this paper, *ℕ*, ℝ^+^, and ℝ* denote the set of all natural numbers, the set of all positive real numbers, and the set of all nonnegative real numbers, respectively.

## 2. TVS-Cone Metric Spaces

**Definition 3 (see [12, 14])**

Let *E* be a topological vector space with its zero vector *θ*. A subset *P* of *E* is called a tvs-cone in *E* if the following are satisfied.

1. *P* is a closed in *E* with a nonempty interior *P*°.
2. *α*, *β* ∈ *P* and *a*, *b* ∈ ℝ*⇒*aα* + *bβ* ∈ *P*.
3. *α*, −*α* ∈ *P*  ⇒  *α* = *θ*.

**Remark 4**

Let *E* be a topological vector space with a tvs-cone *P*. It is clear that *θ* ∈ *P* from Definition 3(2). In addition, it is easy to see that *θ* ∉ *P*°. In fact, pick *α* ∈ *E* − {*θ*}. Then {(1/*n*)*α*} → *θ* and {−(1/*n*)*α*} → *θ* when *n* → *∞*. If *θ* ∈ *P*°, then there is *n* ∈ *ℕ* such that {(1/*n*)*α*, −(1/*n*)*α*}⊆*P*°⊆*P*. By Definition 3(3), (1/*n*)*α* = *θ*. This contradicts that *α* ≠ *θ*. So *θ* ∉ *P*°.

**Definition 5 (see [12, 14])**

Let *E* be a topological vector space with a tvs-cone *P*. Some partial orderings ≤, <, and ≪ on *E* with respect to *P* are defined as follows, respectively. Let *α*, *β* ∈ *E*.

1. *α* ≤ *β* if *β* − *α* ∈ *P*.
2. *α* < *β* if *α* ≤ *β* and *α* ≠ *β*.
3. *α* ≪ *β* if *β* − *α* ∈ *P*°.

**Remark 6**

For the sake of convenience, we also use notations “≥”, “>,” and “≫” on *E* with respect to *P*. The meanings of these notations are clear and the following hold:

1. *α* ≥ *β*⇔*α* − *β* ≥ *θ*⇔*α* − *β* ∈ *P*,
2. *α* > *β*⇔*α* − *β* > *θ*⇔*α* − *β* ∈ *P* − {*θ*},
3. *α* ≫ *β*⇔*α* − *β* ≫ *θ*⇔*α* − *β* ∈ *P*°,
4. *α* ≫ *β*⇒*α* > *β*⇒*α* ≥ *β*.

**Definition 7 (see [10])**

A tvs-cone *P* in a topological vector space *E* is called strongly minihedral if each subset of *E* bounded above has a supremum, equivalently, if each subset of *E* bounded below has an infimum.

In this paper, we always suppose that a tvs-cone *P* in a topological vector space *E* is strongly minihedral.

**Lemma 8**

Let *E* be a topological vector space with a tvs-cone *P*. Then the following hold.

1. If *α* ≫ *θ*, then *rα* ≫ *θ* for each *r* ∈ ℝ^+^.
2. If *α*_1_ ≫ *β*_1_ and *α*_2_ ≥ *β*_2_, then *α*_1_ + *α*_2_ ≫ *β*_1_ + *β*_2_.
3. If *α* ≫ *θ* and *β* ≫ *θ*, then there is *γ* ≫ *θ* such that *γ* ≪ *α* and *γ* ≪ *β*.

**Proof**

(1) Let *α* ≫ *θ*; that is, *α* ∈ *P*°. Then there is an open neighborhood *B* of *α* in *E* such that *B*⊆*P*. If *r* ∈ ℝ^+^, then *rB*⊆*P* from Definition 3(2), where *rB* = {*rβ* : *β* ∈ *B*}. Note that *rα* ∈ *rB* and *rB* is an open subset of *E*. So *rα* ∈ *P*°; that is *rα* ≫ *θ*.

(2) Let *α*_1_ ≫ *β*_1_ and *α*_2_ ≥ *β*_2_. Then *α*_1_ − *β*_1_ ≫ *θ* and *α*_2_ − *β*_2_ ≥ *θ*; that is, *α*_1_ − *β*_1_ ∈ *P*° and *α*_2_ − *β*_2_ ∈ *P*. So there is an open neighborhood *B* of *α*_1_ − *β*_1_ in *E* such that *B*⊆*P*. Write (*α*_2_ − *β*_2_) + *B* = {(*α*_2_ − *β*_2_) + *β* : *β* ∈ *B*}. Note that (*α*_2_ − *β*_2_) + *B* is an open subset of *E*, and (*α*_2_ − *β*_2_)+(*α*_1_ − *β*_1_)∈(*α*_2_ − *β*_2_) + *B*⊆*P* from Definition 3(2). So (*α*_2_ − *β*_2_)+(*α*_1_ − *β*_1_) ∈ *P*°; that is, (*α*_2_ − *β*_2_)+(*α*_1_ − *β*_1_) ≫ *θ*; hence, (*α*_1_ + *α*_2_)−(*β*_1_ + *β*_2_) ≫ *θ*. It follows that *α*_1_ + *α*_2_ ≫ *β*_1_ + *β*_2_.

(3) Let *α* ≫ *θ* and *β* ≫ *θ*; that is, *α*, *β* ∈ *P*°. Then there is *n*_1_, *n*_2_ ∈ *ℕ* such that *α* − ((*α* + *β*)/*n*) ∈ *P*° for all *n* ≥ *n*_1_ and *β* − ((*α* + *β*)/*n*) ∈ *P*° for all *n* ≥ *n*_2_. Put *γ* = (*α* + *β*)/*n*_0_, where *n*_0_ = max⁡{*n*_1_, *n*_2_}. Then *γ* ≫ *θ* from the above (1) and (2). It is clear that *α* − *γ* ∈ *P*° and *β* − *γ* ∈ *P*°; that is, *α* − *γ* ≫ *θ* and *β* − *γ* ≫ *θ*. So *γ* ≪ *α* and *γ* ≪ *β*.

We give the definition of tvs-cone metric, which is very similar to the well-known definition of metric.

**Definition 9 (see [14])**

Let *X* be a nonempty set and let *E* be a topological vector space with a tvs-cone *P*. A mapping *d* : *X* × *X* → *E* is called a tvs-cone metric on *X*, and (*X*, *d*) is called a tvs-cone metric space if the following are satisfied.

1. *d*(*x*, *y*) ≥ *θ* for all *x*, *y* ∈ *X* and *d*(*x*, *y*) = *θ* if and only if *x* = *y*.
2. *d*(*x*, *y*) = *d*(*y*, *x*) for all *x*, *y* ∈ *X*.
3. *d*(*x*, *y*) ≤ *d*(*x*, *z*) + *d*(*z*, *y*) for all *x*, *y*, *z* ∈ *X*.

Note that hemimetric takes values in the extended nonnegative real numbers ([16]). We let *∞* as a possible value of the mapping *d* in the following definition, where *∞* ∉ *E* and the following hold.

- *∞* + *α* = *∞* + *∞* = *∞* for each *α* ∈ *E*.
- *α* ≪ *∞* for each *α* ∈ *E*.

**Definition 10**

Let *X* be a nonempty set and let *E* be a topological vector space with a tvs-cone *P*. A mapping *d* : *X* × *X* → *E*⋃{*∞*} is called a tvs-cone hemimetric on *X*, and (*X*, *d*) is called a tvs-cone hemimetric space if the following (1) and (2) are satisfied.

1. *d*(*x*, *y*) ≤ *d*(*x*, *z*) + *d*(*z*, *y*) for all *x*, *y*, *z* ∈ *X*.
2. *d*(*x*, *x*) = *θ* for all *x* ∈ *X*.

**Proposition 11**

Let (*X*, *d*) be a tvs-cone hemimetric space. Put *B*(*x*, *ɛ*) = {*y* ∈ *X* : *d*(*x*, *y*) ≪ *ɛ*} for *x* ∈ *X* and *ɛ* ≫ *θ*, and put *ℬ* = {*B*(*x*, *ɛ*) : *x* ∈ *X* *and* *ɛ* ≫ *θ*}. Then *ℬ* is a base for some topology on *X*.

**Proof**

It is clear that *X* = ⋃*ℬ*. Let *B*(*x*, *α*), *B*(*y*, *β*) ∈ *ℬ* and *z* ∈ *B*(*x*, *α*)⋂*B*(*y*, *β*). Since *z* ∈ *B*(*x*, *α*), *d*(*x*, *z*) ≪ *α*. Put *γ*_1_ = *α* − *d*(*x*, *z*); then *γ*_1_ ≫ *θ*. We claim that *B*(*z*, *γ*_1_)⊆*B*(*x*, *α*). In fact, if *u* ∈ *B*(*z*, *γ*_1_), then *d*(*z*, *u*) ≪ *γ*_1_; hence, *d*(*x*, *u*) ≤ *d*(*x*, *z*) + *d*(*z*, *u*) ≪ *d*(*x*, *z*) + *γ*_1_ = *α*, and so *u* ∈ *B*(*x*, *α*). Using the same way, we can obtain that there is *γ*_2_ ≫ *θ* such that *B*(*z*, *γ*_2_)⊆*B*(*y*, *β*). By Lemma 8(3), there is *γ* ≫ *θ* such that *γ* ≪ *γ*_1_ and *γ* ≪ *γ*_2_. Let *v* ∈ *B*(*z*, *γ*); then *d*(*z*, *v*) ≪ *γ* ≪ *γ*_1_ and *d*(*z*, *v*) ≪ *γ* ≪ *γ*_2_, so *v* ∈ *B*(*z*, *γ*_1_)⊆*B*(*x*, *α*) and *v* ∈ *B*(*z*, *γ*_2_)⊆*B*(*y*, *β*), and hence *v* ∈ *B*(*x*, *α*)⋂*B*(*y*, *β*). This has proved that *B*(*z*, *γ*)⊆*B*(*x*, *α*)⋂*B*(*y*, *β*). Note that *z* ∈ *B*(*z*, *γ*) ∈ *ℬ*. Consequently, *ℬ* is a base for some topology on *X*. In fact, put *τ* = {*U*⊆*X* : there  is  *ℬ*′⊆*ℬ* such that *U* = ⋃*ℬ*′}; then *τ* is a topology on *X* and *ℬ* is a base for *τ*.

## 3. Superspaces of TVS-Cone Metric Spaces

**Definition 12**

Let (*X*, *d*) be a tvs-cone metric space and let *τ* denote the topology on *X* induced by the tvs-cone metric *d* described in Proposition 11. For an arbitrary nonempty subset *G* of *X*, [*∅*, *G*] and *I*_*G*_ denote the subfamilies {*P* ∈ *𝒫*_0_(*X*) : *P*⊆*G*} and {*P* ∈ *𝒫*_0_(*X*) : *P*⋂*G* ≠ *∅*} of *𝒫*_0_(*X*), respectively.

1. *𝒯*_*ℒ*_ is called the lower topology on *𝒫*_0_(*X*), where *𝒯*_*ℒ*_ is generated by the subbase *ℒ* = {*I*_*G*_ : *G* ∈ *τ*}.
2. *𝒯*_*𝒰*_ is called the upper topology on *𝒫*_0_(*X*), where *𝒯*_*𝒰*_ is generated by the base *𝒰* = {[*∅*, *G*] : *G* ∈ *τ*}.
3. *𝒯*_*𝒱*_ is called the Vietoris topology on *𝒫*_0_(*X*), where *𝒯*_*𝒱*_ is generated by *ℒ* and *𝒰* together.

**Definition 13**

Let (*X*, *d*) be a tvs-cone metric space. For *P*, *Q* ∈ *𝒫*_0_(*X*), put *δ*_*l*_(*P*, *Q*) = inf⁡{*ɛ* ≫ *θ* : *P*⊆*S*(*Q*, *ɛ*)}, *δ*_*u*_(*P*, *Q*) = inf⁡{*ɛ* ≫ *θ* : *Q*⊆*S*(*P*, *ɛ*)}, and *δ*(*P*, *Q*) = inf⁡{*ɛ* ≫ *θ* : *ɛ* ≫ *δ*_*l*_(*P*, *Q*) and *ɛ* ≫ *δ*_*u*_(*P*, *Q*)}, where inf⁡*∅* = *∞*. Then *δ*_*l*_, *δ*_*u*_, and *δ* are tvs-cone hemimetrics on *𝒫*_0_(*X*). Let *𝒯*_*l*_, *𝒯*_*u*_, and *𝒯* denote the topologies on *𝒫*_0_(*X*) induced by *δ*_*l*_, *δ*_*u*_, and *δ* described in Proposition 11, respectively.

1. The topology *𝒯*_*l*_ is called the lower tvs-cone hemimetric topology.
2. The topology *𝒯*_*u*_ is called the upper tvs-cone hemimetric topology.
3. The topology *𝒯* is called the Hausdorff tvs-cone hemimetric topology.

**Remark 14**

It is often preferable to restrict the six topologies *𝒯*_*ℒ*_, *𝒯*_*𝒰*_, *𝒯*_*𝒱*_ *𝒯*_*l*_, *𝒯*_*u*_, and *𝒯* to *𝒦*_0_(*X*), and the relative topologies on *𝒦*_0_(*X*) will still be denoted by *𝒯*_*ℒ*_, and so forth. Also, if *δ*_*u*_ (resp., *δ*_*l*_, *δ*) is restricted to *𝒦*_0_(*X*), then it does not take *∞*.

In this section, we need to use the following notation.

*Notation.* Let (*X*, *d*) be a tvs-cone metric space, *x* ∈ *X*, *P* ∈ *𝒫*_0_(*X*), *K* ∈ *𝒦*_0_(*X*), and *ɛ* ≫ *θ*. Consider the following:

1. *B*(*x*, *ɛ*) = {*y* ∈ *X* : *d*(*x*, *y*) ≪ *ɛ*},
2. *S*(*P*, *ɛ*) = ⋃{*B*(*x*, *ɛ*) : *x* ∈ *P*},
3. *B*_*l*_(*P*, *ɛ*) = {*P*′ ∈ *𝒫*_0_(*X*) : *δ*_*l*_(*P*, *P*′) ≪ *ɛ*},
4. *B*_*u*_(*P*, *ɛ*) = {*P*′ ∈ *𝒫*_0_(*X*) : *δ*_*u*_(*P*, *P*′) ≪ *ɛ*},
5. *C*_*l*_(*K*, *ɛ*) = {*K*′ ∈ *𝒦*_0_(*X*) : *δ*_*l*_(*K*, *K*′) ≪ *ɛ*},
6. *C*_*u*_(*K*, *ɛ*) = {*K*′ ∈ *𝒦*_0_(*X*) : *δ*_*u*_(*K*, *K*′) ≪ *ɛ*},
7. *C*(*K*, *ɛ*) = {*K*′ ∈ *𝒦*_0_(*X*) : *δ*(*K*, *K*′) ≪ *ɛ*}.

**Theorem 15**

Let (*X*, *d*) be a tvs-cone metric space and let *𝒞* be a subset of *𝒫*_0_(*X*). Then the following hold.

1. If *𝒞* is open in the lower topology *𝒯*_*ℒ*_ on *𝒫*_0_(*X*), then *𝒞* is open in the lower tvs-cone hemimetric topology *𝒯*_*l*_ on *𝒫*_0_(*X*).
2. If *𝒞* is open in the upper tvs-cone hemimetric topology *𝒯*_*u*_ on *𝒫*_0_(*X*), then *𝒞* is open in the upper topology *𝒯*_*𝒰*_ on *𝒫*_0_(*X*).

**Proof**

(1) Let *𝒞* be open in the lower topology *𝒯*_*ℒ*_ on *𝒫*_0_(*X*). Without loss of generality, we can assume that *𝒞* is an element in the subbase *ℒ* for the lower topology *𝒯*_*ℒ*_; that is, *𝒞* = *I*_*G*_ for some *G* ∈ *𝒯*. Let *P* ∈ *𝒞*, then *P*⋂*G* ≠ *∅*. Pick *x* ∈ *P*⋂*G*; then there is *ɛ* ≫ *θ* such that *B*(*x*, *ɛ*)⊆*G* since *G* is open in *X*. Let *Q* ∈ *B*_*l*_(*P*, *ɛ*); then *δ*_*l*_(*P*, *Q*) ≪ *ɛ*; that is, *P*⊆*S*(*Q*, *ɛ*). Since *x* ∈ *P*, *x* ∈ *S*(*Q*, *ɛ*), hence *x* ∈ *B*(*y*, *ɛ*) for some *y* ∈ *Q*. Thus, *y* ∈ *B*(*x*, *ɛ*)⊆*G*, which means that *Q*⋂*G* ≠ *∅*. It follows that *Q* ∈ *𝒞*. This proves that *B*_*l*_(*P*, *ɛ*)⊆*𝒞*. So *P* is an interior point of *𝒞* in the lower tvs-cone hemimetric topology *𝒯*_*l*_ and the proof is completed.

(2) Let *𝒞* be open in the upper tvs-cone hemimetric topology *𝒯*_*u*_ on *𝒫*_0_(*X*). Let *P* ∈ *𝒞*; then there is *ɛ* ≫ *θ* such that *B*_*u*_(*P*, *ɛ*)⊆*𝒞*. Note that *S*(*P*, *ɛ*) is open in *X*. So [*∅*, *S*(*P*, *ɛ*)] is open in the upper topology on *𝒫*_0_(*X*). Clearly, *P* ∈ [*∅*, *S*(*P*, *ɛ*)]. On the other hand, if *Q* ∈ [*∅*, *S*(*P*, *ɛ*)], that is, *Q*⊆*S*(*P*, *ɛ*), then *δ*_*u*_(*P*, *Q*) ≪ *ɛ*, and hence *Q* ∈ *B*_*u*_(*P*, *ɛ*)⊆*𝒞*. This has proved that [*∅*, *S*(*P*, *ɛ*)]⊆*𝒞*. Consequently, *𝒞* is an open neighborhood of *P* for the upper topology *𝒯*_*𝒰*_ on *𝒫*_0_(*X*) and the proof is completed.

**Remark 16**

(1) The converses of both (1) and (2) in Theorem 15 are not true (even if (*X*, *d*) is a metric space). Moreover, there is no simple relationship between the Vietoris topology *𝒯*_*𝒱*_ and the Hausdorff tvs-cone hemimetric topology *𝒯* on *𝒫*_0_(*X*), which is similar to (1) or (2) in Theorem 15 (see [16], e.g.).

(2) It is clear that “*𝒫*_0_(*X*)” in Theorem 15 can be replaced by *𝒦*_0_(*X*). Furthermore, we have the better results for the topologies on superspaces *𝒦*_0_(*X*) (see the following).

**Lemma 17**

Let (*X*, *d*) be a tvs-cone metric space. If *K* is a compact subset of *X*, then, for any *ɛ* ≫ *θ*, there is a finite subset *F* of *K* such that *K*⊆*S*(*F*, *ɛ*).

**Proof**

Let *K* be a compact subset of *X* and let *ɛ* ≫ *θ*. Then {*B*(*x*, *ɛ*) : *x* ∈ *K*} is an open cover of *K*; there is a finite subset *F* of *K* such that {*B*(*x*, *ɛ*) : *x* ∈ *F*} covers *K*. It follows that *K*⊆*S*(*F*, *ɛ*).

**Lemma 18**

Let (*X*, *d*) be a tvs-cone metric space. If *K*⊆*U* with *K* compact in *X* and *U* open in *X*, then there is *ɛ* ≫ *θ* such that *S*(*K*, *ɛ*)⊆*U*.

**Proof**

Let *K*⊆*U* with *K* compact in *X* and *U* open in *X*. By Proposition 11, for each *x* ∈ *K*⊆*U*, there is *η*_*x*_ ≫ *θ* such that *B*(*x*, *η*_*x*_)⊆*U*. Put *ε*_*x*_ = (1/2)*η*_*x*_; then *ε*_*x*_ ≫ *θ* from Lemma 8(1). Since {*B*(*x*, *ε*_*x*_) : *x* ∈ *K*} is an open cover of *K* and *K* is compact, there is a finite subset *F* of *K* such that {*B*(*x*, *ε*_*x*_) : *x* ∈ *F*} covers *K*. By Lemma 8(3), there is *ɛ* ≫ *θ* such that *ɛ* ≪ *ε*_*x*_ for each *x* ∈ *F*. We claim that *S*(*K*, *ɛ*)⊆*U*. In fact, let *u* ∈ *S*(*K*, *ɛ*); then there is *y* ∈ *K* such that *u* ∈ *B*(*y*, *ɛ*), that is, *d*(*u*, *y*) ≪ *ɛ*. Furthermore, there is *z* ∈ *F* such that *y* ∈ *B*(*z*, *ε*_*z*_); that is, *d*(*y*, *z*) ≪ *ε*_*z*_. By Lemma 8(2), *d*(*u*, *z*) ≤ *d*(*u*, *y*) + *d*(*y*, *z*) ≪ *ɛ* + *ε*_*z*_ ≪ 2*ε*_*z*_ = *η*_*z*_. It follows that *u* ∈ *B*(*z*, *η*_*z*_)⊆*U*. This has proved that *S*(*K*, *ɛ*)⊆*U*.

**Theorem 19**

Let (*X*, *d*) be a tvs-cone metric space. Then the following hold.

1. The lower topology *𝒯*_*ℒ*_ and the lower tvs-cone hemimetric topology *𝒯*_*l*_ coincide on *𝒦*_0_(*X*).
2. The upper topology *𝒯*_*𝒰*_ and the upper tvs-cone hemimetric topology *𝒯*_*u*_ coincide on *𝒦*_0_(*X*).
3. The Vietoris topology *𝒯*_*𝒱*_ and the Hausdorff tvs-cone hemimetric topology *𝒯* coincide on *𝒦*_0_(*X*).

**Proof**

(1) Assume that *𝒞* is open in the lower hemimetric topology *𝒯*_*l*_ on *𝒦*_0_(*X*). Let *K* ∈ *𝒞*; then there is *ɛ* ≫ *θ* such that *C*_*l*_(*K*, *ɛ*)⊆*𝒞*. Since *K* is compact, by Lemma 17, there is a finite subset *F* of *K* such that *K*⊆*S*(*F*, *ɛ*/2). We write *G*_*x*_ = *B*(*x*, *ɛ*/2) for each *x* ∈ *F* and put *𝒲* = ⋂{*I*_*G*_*x*__ : *x* ∈ *F*}. Note that *B*(*x*, *ɛ*/2) ∈ *𝒯* for each *x* ∈ *F*. It is clear that *K* ∈ *𝒲* and *𝒲* is an element of the base for the lower topology *𝒯*_*ℒ*_ on *𝒦*_0_(*X*). Let *K*′ ∈ *𝒲*. For each *x* ∈ *F*, we claim that *G*_*x*_⊆*S*(*K*′, *ɛ*). In fact, let *y* ∈ *G*_*x*_; then *d*(*x*, *y*) ≪ *ɛ*/2. Since *K*′ ∈ *I*_*G*_*x*__, *K*′⋂*G*_*x*_ ≠ *∅*. Pick *z* ∈ *K*′⋂*G*_*x*_; then *d*(*z*, *x*) ≪ *ɛ*/2; hence, *d*(*z*, *y*) ≤ *d*(*z*, *x*) + *d*(*x*, *y*) ≪ *ɛ*/2 + *ɛ*/2 = *ɛ*; that is, *y* ∈ *B*(*z*, *ɛ*)⊆*S*(*K*′, *ɛ*). This proves that *G*_*x*_⊆*S*(*K*′, *ɛ*). Furthermore, *K*⊆*S*(*F*, *ɛ*/2) = ⋃{*G*_*x*_ : *x* ∈ *F*}⊆*S*(*K*′, *ɛ*). Thus, *δ*_*l*_(*K*, *K*′) ≪ *ɛ*; that is, *K*′ ∈ *C*_*l*_(*K*, *ɛ*)⊆*𝒞*. This proves that *𝒲* ⊂ *𝒞*. It follows that *K* is an interior point of *𝒞* for the lower topology *𝒯*_*ℒ*_ on *𝒦*_0_(*X*). Consequently, *𝒞* is open in the lower topology *𝒯*_*ℒ*_ on *𝒦*_0_(*X*). Combining Remark 16(2), the proof is completed.

(2) Let *𝒞* be open in the upper topology *𝒯*_*𝒰*_ on *𝒦*_0_(*X*). Without loss of generality, we can assume that *𝒞* is an element of the base for the upper topology *𝒯*_*𝒰*_ on *𝒦*_0_(*X*); that is, *𝒞* = [*∅*, *G*]⋂*𝒦*_0_(*X*) for some *G* ∈ *𝒯*. Let *K* ∈ *𝒞*; then *K*⊆*G* and *K* is compact. By Lemma 18, there exists *ɛ* ≫ *θ* such that *S*(*K*, *ɛ*)⊆*G*. If *K*′ ∈ *C*_*u*_(*K*, *ɛ*); then *δ*_*u*_(*K*, *K*′) ≪ *ɛ*; hence, *K*′⊆*S*(*K*, *ɛ*)⊆*G*. It follows that *K*′ ∈ [*∅*, *G*]⋂*𝒦*_0_(*X*) = *𝒞*. Consequently, *C*_*u*_(*K*, *ɛ*)⊆*𝒞*. This has proved that *𝒞* is an open neighborhood of *K* for the upper tvs-cone hemimetric topology *𝒯*_*u*_ on *𝒦*_0_(*X*). Combining Remark 16(2), the proof is completed.

We need to prove that *𝒯*_*𝒱*_⊆*𝒯* and *𝒯*⊆*𝒯*_*𝒱*_. Let *𝒞* be open in the Vietoris topology *𝒯*_*𝒱*_ on *𝒦*_0_(*X*). Without loss of generality, we only need to assume that *𝒞* is an element of the subbase *ℒ* for the lower topology *𝒯*_*ℒ*_ or an element of the base *𝒰* for the upper topology *𝒯*_*𝒰*_. If *𝒞* ∈ *ℒ*, then *𝒞* ∈ *𝒯*_*l*_ by (1). So, for each *K* ∈ *𝒞*, there is *ɛ* ≫ *θ* such that *C*_*l*_(*K*, *ɛ*)⊆*𝒞*. It is clear that *C*(*K*, *ɛ*)⊆*C*_*l*_(*K*, *ɛ*). In fact, if *K*′ ∈ *C*(*K*, *ɛ*), then *δ*(*K*, *K*′) ≪ *ɛ*. Note that *δ*_*l*_(*K*, *K*′) ≤ *δ*(*K*, *K*′) ≪ *ɛ*. So *K*′ ∈ *C*_*l*_(*K*, *ɛ*). It follows that *K* ∈ *C*(*K*, *ɛ*)⊆*C*_*l*_(*K*, *ɛ*)⊆*𝒞*. So *K* is an interior point of *𝒞* for the Hausdorff tvs-cone hemimetric topology *𝒯*. Consequently, *𝒞* is open in the Hausdorff tvs-cone hemimetric topology *𝒯* on *𝒦*_0_(*X*). By a similar way, if *𝒞* ∈ *𝒰*, then *𝒞* is open in the Hausdorff tvs-cone hemimetric topology *𝒯* on *𝒦*_0_(*X*). This has proved that *𝒯*_*𝒱*_⊆*𝒯*. Conversely, let *𝒞* be open in the Hausdorff tvs-cone hemimetric topology *𝒯* on *𝒦*_0_(*X*). Then, for each *K* ∈ *𝒞*, there is *ɛ* ≫ *θ* such that *C*(*K*, *ɛ*)⊆*𝒞*. We claim that *C*_*l*_(*K*, *ɛ*/2)⋂*C*_*u*_(*K*, *ɛ*/2)⊆*C*(*K*, *ɛ*). In fact, if *K*′ ∈ *C*_*l*_(*K*, *ɛ*/2)⋂*C*_*u*_(*K*, *ɛ*/2), then *δ*_*l*_(*K*, *K*′) ≪ *ɛ*/2 and *δ*_*u*_(*K*, *K*′) ≪ *ɛ*/2. So *δ*(*K*, *K*′) ≤ *δ*_*l*_(*K*, *K*′) + *δ*_*u*_(*K*, *K*′) ≪ *ɛ*/2 + *ɛ*/2 = *ɛ*; hence, *K*′ ∈ *C*(*K*, *ɛ*). This proves that *C*_*l*_(*K*, *ɛ*/2)⋂*C*_*u*_(*K*, *ɛ*/2)⊆*C*(*K*, *ɛ*). By (1) and (2), *C*_*l*_(*K*, *ɛ*/2) and *C*_*u*_(*K*, *ɛ*/2) are open in *𝒯*_*ℒ*_ and *𝒯*_*𝒰*_, respectively. It follows that *C*_*l*_(*K*, *ɛ*/2)⋂*C*_*u*_(*K*, *ɛ*/2) is open in *𝒯*_*𝒱*_. So *K* is an interior point of *𝒞* for the Vietoris topology *𝒯*_*𝒱*_ on *𝒦*_0_(*X*). Consequently, *𝒞* is open in the Vietoris topology *𝒯*_*𝒱*_ on *𝒦*_0_(*X*). This has proved that *𝒯*⊆*𝒯*_*𝒱*_.
